# The *Triticeae CBF* Gene Cluster—To Frost Resistance and Beyond

**DOI:** 10.3390/cells12222606

**Published:** 2023-11-11

**Authors:** Giovanni Caccialupi, Justyna Milc, Federica Caradonia, Muhammad Fazail Nasar, Enrico Francia

**Affiliations:** Department of Life Sciences, University of Modena and Reggio Emilia, Via Amendola 2, 42122 Reggio Emilia, Italy; justynaanna.milc@unimore.it (J.M.); federica.caradonia@unimore.it (F.C.); muhammadfazail.nasar@unimore.it (M.F.N.); enrico.francia@unimore.it (E.F.)

**Keywords:** abiotic stress, *Triticeae*, *CBF* transcription factors, cold acclimation, frost tolerance, drought tolerance

## Abstract

The pivotal role of *CBF*/*DREB1* transcriptional factors in *Triticeae* crops involved in the abiotic stress response has been highlighted. The CBFs represent an important hub in the ICE-CBF-COR pathway, which is one of the most relevant mechanisms capable of activating the adaptive response to cold and drought in wheat, barley, and rye. Understanding the intricate mechanisms and regulation of the cluster of *CBF* genes harbored by the homoeologous chromosome group 5 entails significant potential for the genetic improvement of small grain cereals. *Triticeae* crops seem to share common mechanisms characterized, however, by some peculiar aspects of the response to stress, highlighting a combined landscape of single-nucleotide variants and copy number variation involving *CBF* members of subgroup IV. Moreover, while chromosome 5 ploidy appears to confer species-specific levels of resistance, an important involvement of the *ICE* factor might explain the greater tolerance of rye. By unraveling the genetic basis of abiotic stress tolerance, researchers can develop resilient varieties better equipped to withstand extreme environmental conditions. Hence, advancing our knowledge of *CBFs* and their interactions represents a promising avenue for improving crop resilience and food security.

## 1. *Triticeae* Crops and Abiotic Stress

### 1.1. Triticeae as Staple Food and Adaptable Crops

The Green Revolution had been able to meet the demand for food, reducing world hunger among the growing population (from 2.519 billion in 1950 to 4.435 billion in 1980) thanks to an unprecedented increase in crop yield and agricultural production [1,2]. New irrigation techniques, massive use of fertilizers and plant protection products, mechanization, crop breeding, and adoption of improved varieties were the determining factors in the observed increase in productivity [3,4,5]. Cereal crops, in particular, saw significant improvement, with yields tripling despite a small increase in arable land [6,7]. However, besides the positive effects, the excessive agricultural intensification created the conditions for the rise of environmental problems such as pollution, soil degradation, and loss of genetic diversity [8,9]. For example, in many breeding programs, genotypes were selected for the high-input systems driving gene pool erosion, especially for the alleles responsible for adaptation to the environment [10,11,12]. However, new issues emerged: yield seems to have reached a plateau and a contraction of genetic diversity has been observed [13,14]; as a result, the adaptation to biotic and abiotic stresses of cereal crops has been reduced [15,16,17,18,19]. In a scenario where the population is still growing (based on UN estimations, planet Earth will be populated by 8.5, 9.7, and 10.9 billion people by 2030, 2050, and 2100, respectively [2]), one of the goals of the global food production system is to provide higher yields and food quality while reducing, however, environmental pollution [6]. Furthermore, extreme weather conditions, reduction of arable lands, and increasing demand of fertilizers and irrigation water are putting the crops cultivation in open fields under stress conditions, significantly affecting agricultural production on all continents [20]. A novel approach is required to cope with the climate issue. Crop breeding programs need to develop new genotypes with a higher adaptation to weather fluctuations [21,22] and contribute to global food security [23].

The *Triticeae* tribe, a grass tribe of the *Poaceae* family that includes cultivated wheats (durum wheat *Triticum turgidum* L. ssp. *durum* Desf., bread wheat *Triticum aestivum* L.), barley (*Hordeum vulgare* L.), and rye (*Secale cereale* L.), is by far the most important source of energy and nutrients worldwide [24,25]. For example, wheat and barley together were the most cultivated herbaceous crops in the world in 2021, with a harvested area of 220 and 48 million hectares and a total grain production of 770 and 145 million tons, respectively [26]. Rye is an important crop for Northern and Eastern European countries, with a harvested area of 3.5 million hectares and a total production of 11 million tons [26]. The *Triticeae* tribe comprises about 350 species, including the so-called minor cereals such as triticale, spelt emmer, and einkorn wheats, poulard, polish, and khorasan wheats [27].

Temperate grass species are characterized by winter growth habits (WH) in their natural environments [28,29]. The two key traits of WH genotypes are the vernalization requirement and the cold acclimation. Vernalization is defined as the induction of flowering after prolonged exposure to cold. Moreover, *Triticeae* are usually classified as long-day (LD) plants because most varieties flower earlier when exposed to longer days. This mechanism synchronizes plants to flower after cold, harmful temperatures in the wintertime [30,31]. The cold acclimation is the ability of the crop to adapt to cold temperatures and then survive frost events [32]. The winter habit (WH) genotypes are usually sown in winter due to their higher productivity. In Mediterranean climates, sowing is performed in the autumn to take advantage of the rainiest seasons, and the plants are harvested during the drier summer. Winter habit is a limiting factor in the widespread cultivation of *Triticeae* in environments where winter is too cold to survive or too warm to satisfy the vernalization requirement [33]. To overcome this limit, spring habit (SH) and facultative habit (FH) genotypes were selected for their lack of vernalization requirements [34,35]. SH genotypes are sown in spring, whereas FH genotypes can be alternatively sown either in autumn or spring. Most SH cultivars are frost-prone and, due to a shorter crop cycle, may be exposed to drought. For these reasons, in the last few years, FH genotypes are gaining more and more interest since they show a high level of frost tolerance (FT) and do not require vernalization [36,37]. The *Triticeae* crops are thus adaptable to several environments, ranging from sub-arctic to tropical climates, allowing their cultivation across a wide geographical area [38,39], even if the highest yields are achieved in temperate regions [40].

### 1.2. Cold and Drought Issues for Triticeae in the Climate Change Era

Abiotic stresses are described as environmental conditions that can impact a plant’s growth and production [41]. Temperate cereals usually experience frost during the vegetative/tillering phase, whereas drought usually negatively influences the flowering, heading, and ripening phases. Extreme temperatures and drought are associated with plant cell dehydration and represent some of the major issues for agronomical and global food security in *Triticeae* [12,42]. Frost damage is caused by ice formation in intercellular spaces, resulting in a drop in water potential [43], influencing root water uptake and photosynthesis [44]. Drought stress affects plant growth differently depending on the phenological phase [45]; early events reduce stomatal conductance, transpiration, and CO_2_ assimilation, affecting tiller formation, while water deprivation during the reproductive phase reduces grain number and size [46]. In some environments, a combination of frost and drought (the so-called harsh winter) may happen, causing a severe reduction in enzymatic activities and membrane disintegration, leading to stunted growth and compromising yield [47,48].

The autumn, winter, and spring seasons are becoming more and more variable nowadays, with a higher alternation between warm-dry and cold-rainy periods with extreme frost episodes [49,50,51]. This alternation may putatively cause an overlapping of low temperature and water deprivation in regions—such as the Mediterranean basin—where prolonged droughts followed by/overlapped with cold and rain events become more frequent, with consequences still unclear for crops [52,53]. Moreover, based on the United States Drought Monitor and JRC European Drought Observatory, data collected since 2011 reveal that drought in fall-winter and during early spring has been increasing in temperate areas where *Triticeae* crops are manly cultivated [54,55]. Furthermore, the warmer temperatures in the Arctic pole might affect mostly the northern hemisphere, increasing the severity of winter and frost events in early spring [50,56,57]. The climate fluctuations in temperate regions are impacting the crop cycle and the phenological responses of barley and wheat crops [25,58]. The alternation of warm and cold periods might induce a de-acclimatation process in the WH genotypes, reducing their frost tolerance and producing serious yield losses [59,60]. Additionally, drought stress, which occurs due to a reduced amount of rain and a lower level of soil moisture during the sowing period (autumn/early winter) and in the initial phenological stages, can decrease the rate of germination and early seedling development [61]. Furthermore, the vernalization requirement may not be totally satisfied, resulting in delayed flowering and exposing the plants to a different incidence of biotic and abiotic stresses [62]. Even spring genotypes are not immune to this problem, as the alternation of drought stress and late frost events can compromise germination and development [63,64]. Moreover, SH genotypes may face an increased risk of heat wave exposure during the grain filling phase [65,66].

Due to the multitude of factors involved in climate change, a prediction of yield loss is complex to assess. Several authors have reported that a reduction in the yield of barley and wheat is linked to the geographical area and the consequent impact that climate change has on that specific region [25,67,68]. For example, a study on the impact of climate change in a period between 1981 and 2009 on wheat production in China stated that due to a 1 °C increase, the yield was reduced by 1 to 10% [58]. As far as growth habits are concerned, Gammans and colleagues [69] analyzed the effect of a wide range of climate models and emissions scenarios on winter/spring barley and wheat yield reductions in France for the period 2037–2065. Similarly, Cammarano et al. [70] stated that climate change could impact the yield by up to 9% for barley genotypes with different vernalization requirements cultivated in the Mediterranean basin.

**Figure 1:**
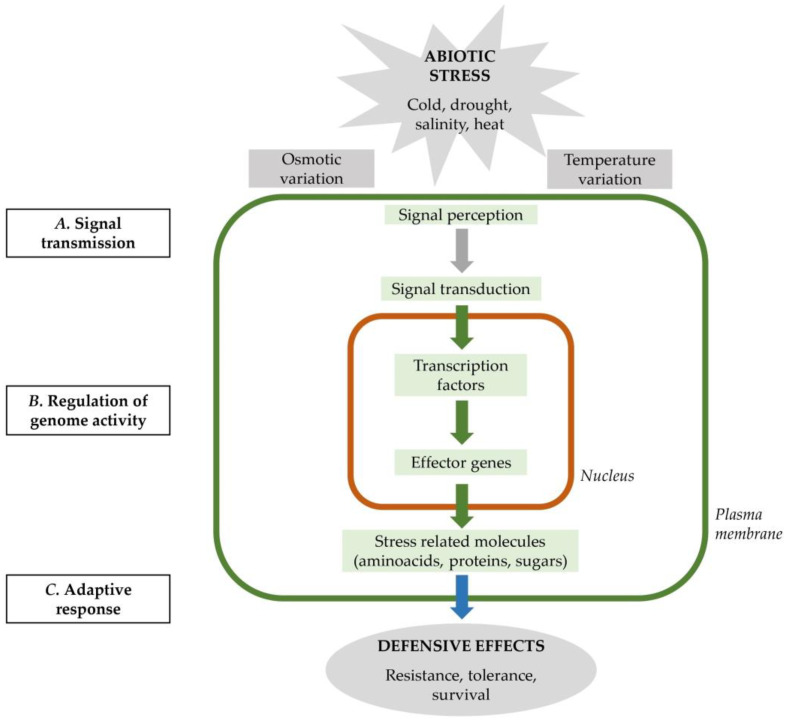
Simplified model of cellular response to temperature and osmotic variations. Starting from perception and transduction of the signal (A), stresses can directly cause physical or chemical changes to biomolecules in the plant cell, triggering a cellular response [79]. The cellular response is driven by signaling events involving hormones, secondary messengers (ROS and Ca^2+^) and regulatory proteins (BON, NGA, and kinase) [80,81]. A complex regulation of genome activity is initiated in the nucleus (B), stress signaling triggers changes in gene transcription and RNA processing [41]. Transcription factors interact with cis-acting elements, which are present in the promoter region of several stress-related genes, also known as effector genes [82]. Key transcriptional regulators target regulons of effector genes that lead to the synthesis of protein in the mitochondria (C). Effector genes encode proteins that alter the plant physiology, producing stress-related molecules, leading to the adaptive response [83]. The final result is an increased ability of the cell, tissue, and plant to cope with and respond effectively to the stresses (modified from [41,84]).

Numerous studies have been undertaken in recent years to elucidate the molecular basis of adaptation process in *Triticeae* cereals for the development of new improved varieties (in Figure 1, we present a simplified model of the cellular adaptative response to osmotic and temperature variations). The crucial role of genetic resources on vernalization response (*VRN-1*, *VRN-2*, and *VRN-3*) and frost tolerance (*FR-1* and *FR-2*) has already been defined by multiple authors [71,72,73,74,75]. *FR-2* is known to encompass a cluster of C-repeat binding factors (*CBF*) genes that are involved in cold acclimation and frost tolerance. In addition, recent reports suggest that they are also involved in drought response [76,77,78].

Maintaining yield performance under various unfavorable conditions is the main concern for breeders regarding the genetic improvement of resistance/tolerance to abiotic stresses. Starting with a comprehensive description of the *CBF* gene cluster locus structure and function, this review aims to better understand the complex signal transduction pathway(s) resulting in the abiotic stress tolerance of *Triticeae*. The availability of different linkage maps and genomic resources, such as transcriptomes and whole genome sequences, can boost the efficacy of breeding programs for wider climatic adaptability and stress tolerance. Thus, our aim is to describe the pivotal role of *Triticeae* CBF transcriptional activators in frost tolerance and to show their putative role in drought tolerance. Integration of omics with substantial trait variation existing in genetic resources would pave the way for cereal crop improvement against abiotic stresses.

## 2. *CBF* Gene Cluster and Its Central Role in Response to Frost and Drought

### 2.1. C-Repeat Binding Factors and Cluster Organization in Triticeae

C-repeat binding factors, or dehydration responsive element (*CBF*/*DREB1*), are a larger subfamily of transcription factors that belong to the APETALA2/ethylene-responsive element binding factor (AP2/ERF) protein family and are induced/activated in response to osmotic stresses such as cold or drought. The AP2/ERF domain binds to the C-repeat/dehydration responsive elements (CRT/DRE) in the promoter region of a variety of genes involved in the abiotic stress response, also known as “CBF’s regulon.” These genes protect against the adverse effects of losing water caused by frost and drought with the biosynthesis of osmoprotectant proteins, carbohydrate metabolism-related activity, and sugar transport [85,86,87]. Among these, cold-regulated genes (*COR*) are the most important family, including late embryo abundant proteins (*LEA*), low-temperature-induced (*LTI*), cold-inducible (*KIN*), responsive to desiccation (*RD*), early dehydration-inducible (*ERD*), and the dehydrin (*DHN*) genes [88,89,90,91]. The distinctive element of *CBFs* within the AP2/ERF family is the specific “CBF signature” flanking the AP2 domain [86,92].

*CBF1* was the first *CBF* gene isolated and characterized by Stockinger and colleagues in *Arabidopsis thaliana* [93]. Subsequently, other important works discovered the *CBF* family and its role in the model plant [94,95] and then in other 54 genera: 31 dicotyledons, 23 monocotyledons, and 13 woody species [96,97,98,99,100]. In *Poaceae*, multiple elements of the family were isolated and characterized, either in chilling-sensitive (e.g., rice and maize) or frost-tolerant species (e.g., wheat, barley, and rye) [96,101,102,103]. The *CBF* genes are characterized by short, mono-exon coding sequences (average length 700 bp) with no introns [96,104,105]. Interestingly, Shi et al. [91] performed a phylogenetic analysis and found that the *CBF* gene structure is remarkably conserved across various species (monocots/dicots), independently of their degree of frost tolerance. As reported by Campoli et al. and Badawi et al. [106,107], *CBF* genes are classified into four phylogenetic groups, each with two or more sub-groups. Some elements of the *CBF* gene family are scattered along the genome, while others, more frequently, are organized in clusters of tandemly duplicated genes on the long arm of homoeologous chromosome group 5 of *Triticeae* [74,108,109,110]. The cluster of *CBF* genes has been shown to coincide with a QTL for frost tolerance, namely *Frost Resistance 2* (*FR-2*) in barley (*FR-H2*), diploid (*FR-A^m^2*) and polyploid wheats (*FR-A2* and *FR-B2*), and rye (*FR-R2*) [74,110,111]. In *Triticeae* crops, beside *FR-2*, part of the phenotypic variation for frost tolerance is attributed to another QTL located about 25–30 cM apart from *FR-2* on the long arm of homoeologous chromosome group 5: *Frost Resistance 1* (*FR-1*). This locus was identified by Hayes et al. in 1993 and Galiba et al. in 1995 [109,112] in barley and wheat, respectively, and reported to co-segregate with *VRN-1*, the vernalization requirement gene [74], whose expression leads the plant to become competent for flowering [113].

**Figure 2:**
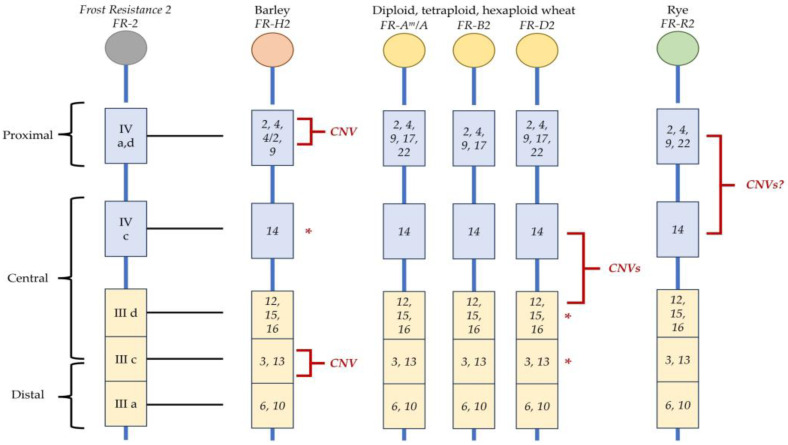
Model of the structural organization of the *CBF* cluster at *Frost Resistance-2* in *Triticeae* genomes. The locus is depicted in proximal, central, and distant parts, highlighting different phylogenetic classifications in *CBF* subgroups. Pale blue boxes—*CBFs* of groups IV; yellow boxes—*CBFs* of III groups; CNV—copy number variation; CNVs?—CNV still not confirmed; red asterisks—SNV (single nucleotide variation). As far as the copy number variations in barley are concerned, multiple copies of the *HvCBF2A-HvCBF4B* genomic segment were identified in the *Frost Resistance-2* locus [121,122]. CNV at *CBF14* in the winter panel was associated with higher expression levels in tolerant haplotypes [123]. Two insertion/deletions and ten single nucleotide polymorphisms were identified for *TaCBF-A12* and *TaCBF-A15* [124]. In rye, the genome assembly [114] revealed CNV for 4 members of CBF Group IV between tolerant and resistant varieties; however, this data has not been confirmed yet.

The number of *CBF* genes identified in *Triticeae* species has been increasing during the last two decades, with novel studies being performed and new genomic data being obtained. The first comprehensive studies reported at least 15 *CBF* genes present in each of the A, B, and D genomes in wheat [107] and 20 in barley [92]. In rye, only 12 *CBF* genes were initially identified [106,114]. Up to date, the estimated number of *CBF* orthologs harbored only at *FR-2* is 13, 54, and 21, for barley, wheat, and rye, respectively [115]. *Triticeae CBFs* can be classified into groups sharing similar structural characteristics and a common phylogenetic origin. Six of these groups, i.e., IIIc, IIId, IVa, IVb, Ivc, and Ivd, had been identified only in the subfamily *Pooideae*, suggesting the recent adaptation of *CBFs* to temperate habitats [107]. Figure 2 shows a simplified model of the *FR-2* cluster organization in *Triticeae*.

Barley *CBF*s were classified by Skinner et al. [92] into three phylogenetic clades: HvCBFI, HvCBFIII, and HvCBFIV. Seven *CBF* genes of the HvCBFI clade are widespread across the genome, while *FR-H2* encompasses 13 *CBF*s genes of the HvCBFIII and HvCBFIV clades organized in a single cluster [116]. The cluster is divided into three different portions: proximal (*HvCBF 2*, *4*, and *9*), central (*HvCBF 3*, *12*, *13*, *14*, *15*, *16*), and distal (*HvCBF 6* and *10*). *HvCBF* genes in *FR-H2* cluster are surrounded by non-coding sequences enriched in multiple repetitive elements [117].

Thirteen *TmCBF* were described in *Triticum monococcum* L.; eleven of them were mapped on *FR-A^m^2*, while *TmCBF15* and *TmCBF18* were mapped on chromosomes 7A^m^ and 6A^m^, respectively [118]. Vágújfalvi et al. [110] attributed the locus for FT to chromosome 5A, and subsequently Knox and colleagues [119] divided the *FR-A2* locus into: proximal (*CBF 2*, *4*, *9*, and *17*), central (*CBF 12*, *14*, and *15*), and distal (*CBF 3*, *10*, *13*, and *16*).

The genome of hexaploid wheat encodes 65 *TaCBF*s [120], 27 of which are paralogs with 1–3 homoeologous A, B, and D copies [120]. As reported by The International Wheat Genome Sequencing Consortium (IWGSC) [115], 54 *TaCBF*s are located on chromosome Group 5: 17 genes on 5A, 19 on 5B, and 18 on 5D chromosomes. Other *TaCBF*s are located on homoloegous chromosomes 6 (A, B, and D).

Rye encodes 21 *ScCBF*s, most of which reside at *FR-R2* and were mapped to chromosome 5RL [106,107,111].

### 2.2. Role of the ICE-CBF-COR Pathway in Cold Acclimation

In winter cereals, cold acclimation, also known as “hardening”, has the vital function of protecting the crown and young leaves from ice damage [125]. Even after a severe stress episode, if the crown and young leaves survive, the plant maintains the potential to restore from tillering nodes [126]. This peculiarity is linked to the ability of the meristematic tissue to survive thanks to the physiological phenomenon of cold acclimation [127]. Phenolic compounds, sugars, soluble proteins, new enzyme isoforms, proline and organic acids, modification of the fatty acid composition in the phospholipid membrane, and higher levels of antioxidants are all proactive compounds connected to the reduction of frost damage [87,125,128,129].

**Figure 3:**
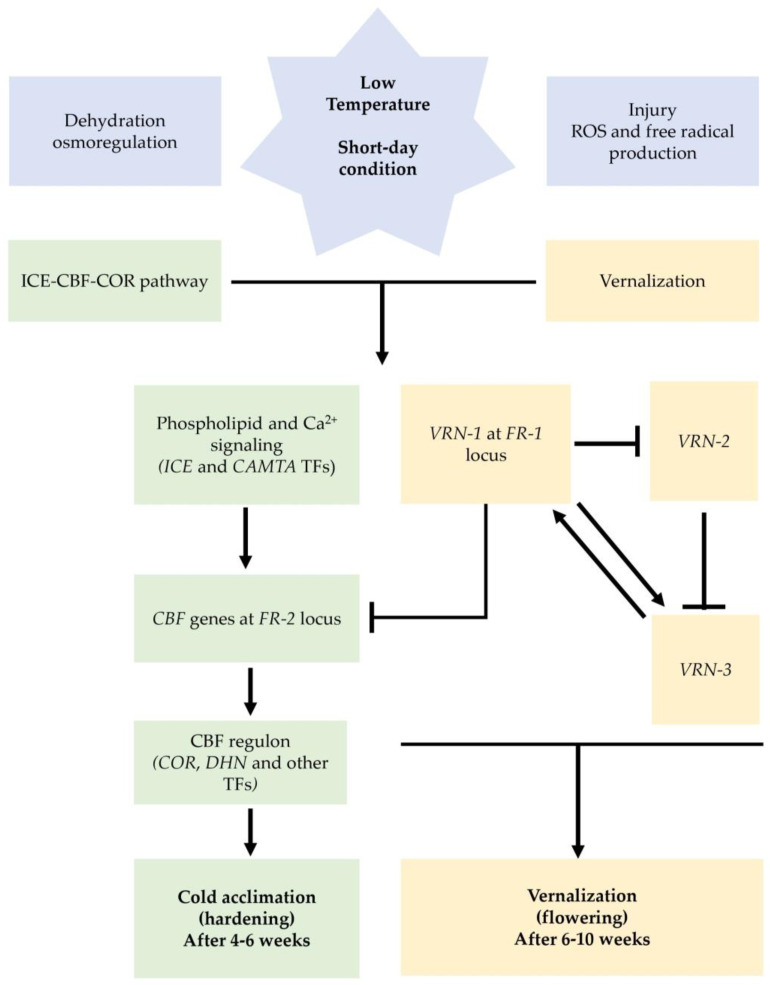
Schematic diagram connecting cold acclimation and vernalization as they are thought to occur in WH genotypes (taken as reference). Black arrows—gene induction; black lines with blunt ends—gene repression; green boxes—the ICE-CBF-COR pathway; yellow boxes—the vernalization requirement genes. The CBF pathway (green) responds to short cold exposure and short-day conditions. While the low-temperature receptors are unknown, the Ca^2+^ signal initiates the pathway through membrane rigidification and Ca-sensor binding [144,145]. Inducers of *CBF*-gene expression (*ICE*) and Calmodulin-binding transcription activators (*CAMTA*) transcription factors up-regulate *CBF* genes at *FR-2* locus [78,146,147]. Consequently, *CBF* genes up-regulate the effector genes and after 4–6 weeks of cold acclimation, crops can withstand sub-zero temperatures [141,142]. Vernalization (yellow) is governed by three genes: *VRN-1*, *VRN-2*, and *VRN-3*. *VRN-1* acts as an AP1-like MADS-box transcription factor, triggering the shift from vegetative to reproductive phases and contributing to cold acclimation and frost tolerance [73,157]. During autumn’s long/neutral days, *VRN-2* is highly expressed, repressing *VRN-3*, the flowering induction gene, and limiting *VRN-1* expression [161,165]. Exposure to cold activates *VRN-1*, leading to *VRN-2* down-regulation and *VRN-3* release from repression. After extended cold exposure, *VRN-1*’s expression reaches a threshold, inducing the transition to flowering, aided by long-day conditions and *PPD-1/PPD-2* activation. The connection between *CBF* and the vernalization pathway is due to higher *VRN-1* levels, which down-regulate *CBF* genes, reducing frost tolerance [31,73]. VRN-1 can bind *CBF* gene promoters, potentially reducing their transcription. Vernalization time varies based on genotype origin and environmental conditions, spanning 6 to 10 weeks at temperatures from 6 °C to 2 °C under short days [31,141,142,168].

In winter barley, wheat, and rye, cold acclimation occurs only in the vegetative phase, and it has two different signaling pathways: abscisic acid or ABA-dependent (ABA pathway) and ABA-independent (also known as the ICE-CBF-COR pathway) [130]. Although the ABA and CBF signal transmissions were considered distinct from each other, recent studies suggest a cross-talk between these two pathways [131] (Figure 3).

In short-day conditions, the ICE-CBF-COR pathway is promptly activated after a brief exposure to low, non-harmful temperatures [132,133], and the *CBF* gene has a pivotal role in the coordination of the acclimation processes [134]. In Arabidopsis, a marked increase in *CBF* transcript levels was observed 15 min after cold exposure, followed by up-regulation of the effector genes about 2 h later [32,135]. On the other hand, in wheat and barley, an increase in *CBF* transcript levels was observed 4–12 h later after the cold exposure [75,136,137]. The gene induction relies on a temperature threshold dependent on the species and occurs in a 10 °C to 12 °C range in winter barley, wheat, and rye [138,139]. The result of the ICE-CBF-COR pathway cascade is the activation of the effector genes that modify the plant metabolism, conferring frost tolerance [140]. The temperature must be below 10 °C for 4–6 weeks in short-day conditions to complete the adaptive response in *Triticeae* [141,142]; once the process is completed, crops can withstand freezing at −7/12 °C for barley, −9/18 °C for wheat, and −18/−30 °C for rye [132,143].

Interestingly, no receptors receiving the low temperature signal have been identified so far [128]. The ICE-CBF-COR pathway is activated by an increase in intracellular Ca^2+^ concentration by either rigidification of the plasma membrane or ligand-activated channels. After calcium influx into the cytosol and its binding by Ca-sensors (such as calmodulins), a signal cascade based on calcium-binding proteins (CBPs) is initiated to target the *ICE* (inducers of *CBF*-gene expression) transcription factors that up-regulate the *CBF* genes [144,145]. *ICE* transcription factors belong to the MYC family and MYC subfamily of bHLH (basic helix–loop–helix) [78] and are known as positive *CBF* expression regulators, considered to act upstream of the low-temperature signaling pathway [78,146,147].

**Figure 4:**
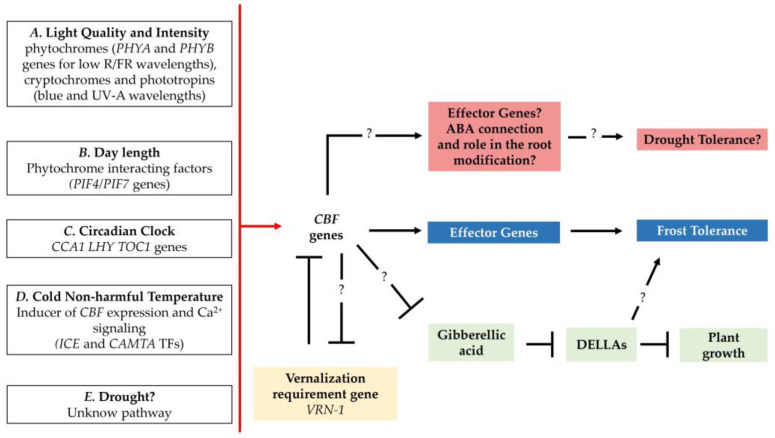
Outline of the integration of environmental stimuli that putatively influence the action of *CBF* genes. White boxes—environmental stimuli that influence (induce or repress) *CBF* genes; colored boxes—the cascade or putative cascade pathways activated by *CBF* genes; black arrows—induction or up-regulation of gene expression; black lines with blunt ends—repression of gene expression; ?—hypothetical interactions still not proven in *Triticeae* crops. Letters in bold—stimuli that influence the CBF expression. (A)—Hypothetical model for the phytochrome-dependent *CBF14* regulation in barley and wheat suggested by Novák et al. [155]. Under white light illumination, *HvCBF14* is repressed by the active form of Phytochrome B (*phyB*), and the expression of Phytochrome A (*PHYA*) is inhibited by light and by *phyB*. Under supplemental FR illumination (low R:FR ratio), most of the phyB proteins are in inactive form and *HvCBF14* or *PHYA* are released under repression. The active form of *phyA* may then enhance the expression of *HvCBF14*. The original model was proposed by Wang et al. [233] for tomato plants. (B)—In Arabidopsis, Phytochrome-interacting factors *PIF4* and *PIF7* are involved in the photoperiodic regulation of *AtCBF2*. In long-day conditions, *PIF4* and *PIF7* down-regulate the *CBF* pathway [150]. (C)—Circadian-clock-associated1 (*CCA1*) and late elongated hypocotyl (*LHY*) are positive regulators of *CBF* expression and plant freezing tolerance [148]. The *CBF* expression peak was observed before the sunset [149]. No circadian-clock-component was found in the promoter region, suggesting an intermediate role by other genes [117]. (D)—Inducers of *CBF*-expression *ICE* and Calmodulin-binding transcription activators *CAMTA* transcription factors are the *CBF* genes expression promoter under the cold stimuli [81,147]. (E)—Concerning the drought, in barley, it has been shown that HvCBF1- and HvCBF3-subgroup proteins bind the DRE/CRT motif promoter either at warm or low temperatures, and several drought responsive genes related to DRE/CRT motif are present in their promoter regions [148]. Nevertheless, which *CBF* genes and which pathway are still unknown. In colored rectangles, the *CBF*’s cascade pathways. Red rectangles—the role of the *CBF* genes in the drought tolerance is still unclear; however, it might have a putative connection with the ABA pathway that induces root modification [94,234]. Green rectangles—*CBF* genes have a role in connecting plant hormones like gibberellic acid and DELLA proteins, increasing freezing tolerance and showing stunted growth and delayed flowering [235,236]. This mechanism was established in Arabidopsis (for a recent review, see [237]); however, there have been no reports for *Triticeae*. Yellow rectangle—for the vernalization, *VRN-1* can bind *CBF* gene promoters, reducing their transcription [72]; however in the first phase of cold acclimation, the CBF protein may have a putative role to down-regulate *VRN-1*.

In addition, as shown in Figure 4, temperature variation is not the only environmental stimulus influencing the expression of the *CBFs*; also, circadian rhythms and light characteristics (i.e., quality and quantity) have been reported to be involved in cold acclimation [133]. For example, recent studies showed that the expression of some barley *HvCBF* genes (*HvCBF2A*, *HvCBF4B*, *HvCBF6*, and *HvCBF14*) is regulated by the circadian rhythm and day length [133,148,149]. In warm conditions, *CBF* genes show high expression late in the afternoon and continue to decrease early in the night [148]. The peak of expression is 8–12 h after the dawn, either in short- or long-day conditions. However, the amplitude of the peaks is wider in short-day compared to long-day conditions [150]. This peak does not coincide with the coolest period of the day, but it may be functional for the preparation of the cell for the subsequent cold of the night [107]. The circadian clock regulates the expression of several genes. The G-Box-like motifs are necessary for transcriptional regulation by the circadian pseudo-response regulators binding basic helix–loop–helix transcription factors [151]. Other environmental stimuli are the light spectra and intensity; several works have elucidated that the variation of light spectra and light intensity might modulate the expression of *CBF* genes and also increase frost tolerance [90,152,153,154,155,156].

The vernalization process is controlled by three major genes: *VRN-1*, *VRN-2*, and *VRN-3* [73,157]. *VRN-1* is a flowering promoter that was shown to be an AP1-like MADS-box transcription factor, whose expression leads the plant to the transition from the vegetative to the reproductive phase [158,159]. Moreover, it was also proven to be involved in cold acclimation and frost tolerance [71]. *VRN-2* is a dominant flowering repressor down-regulated by vernalization treatment and includes two tandem zinc finger-CCT domain genes (ZCCT1 and ZCCT2) [31,160]. *VRN-3*, the main integrator of the photoperiod and vernalization signals that lead to the transition of the apical meristem [161], is homologous to the flowering integrator FLOWERING LOCUS T gene in Arabidopsis [162,163]. Due to their diploid nature, WH barleys can be considered a model for vernalization in Triticeae crops [24]. *VRN-H2* is expressed in long and neutral day conditions [164]. In autumn, when plants are still in the seedling stages, *VRN-H2* is highly expressed and represses the *VRN-H3*, which is the flowering induction gene [161,165]. The repression of *VRN-H3* also limits the expression of *VRN-H1* [158,159]. Exposure to cold temperatures activates *VRN-H1* and results in the down-regulation of *VRN-H2* and, consequently, the release of *VRN-H3* from repression [31,73]. After prolonged cold exposure, the expression level of *VRN-H1* reaches a threshold necessary to induce the transition phase, up-regulating *VRN-H3*, and initiating the flowering process [165]. Exposure to long-day conditions mediated by the photoperiod genes *PPD-H1* and *PPD-H2* is also necessary [53].

The expression of *VRN-H1* changes in function of the plant growth habit; as mentioned above, in winter genotypes, the expression of the recessive *vrn-h1* allele is induced by prolonged periods of cold [166,167]. The quantity of time under cold and short-day conditions necessary to satisfy the vernalization requirements varies with the geographical origin of the genotype and the environmental condition, changing from 6 to 10 weeks of temperatures in a range between 6 °C and 2 °C under short-day conditions [31,141,142,168]. In spring genotype, the dominant *Vrn-h1* allele has a constitutive high expression that rapidly induces the transition [169]. The vernalization in wheat is more complex compared to barley due to the presence of three homoeologous *VRN-A1*, *VRN-B1*, and *VRN*-*D1* loci mapped on the long arm of chromosome group 5 [170], with the major effect of *VRN-A1* in determining the growth habit [171].

The interaction between *VRN-1/FR-1* and *FR-2* (*CBFs*) has also been demonstrated [172]; *VRN-H1* can bind promoter regions of the *CBF* genes, inducing a reduction of their transcription levels; nevertheless, the mechanism is still not fully understood [117,173].

However, a question remains: how does the ICE-CBF-COR pathway confer frost tolerance?

### 2.3. FR-2 in Barley—A Synergistic Action of CNV and HvCBF14?

The efforts to identify the molecular mechanisms underlying *FR-2* in *Triticeae* crops were based on integration studies on structural and functional aspects of the locus (Figure 2). Several barley genotypes have been sequenced, and a pan-genome has been assembled [174]. Thanks to this data, *FR-H2* was studied in different frost-prone and tolerant genotypes to evaluate the *CBFs* position in the cluster, the variability in the structure, the *CBF* coding sequences, and the promoter regions [24,116,175,176,177].

Initially, four *HvCBF* genes (*HvCBF3*, *HvCBF6*, *HvCBF9*, and *HvCBF14*) have been selected as candidate genes due to the presence of homologs in other *Triticeae* already reported to be involved in cold resistance [107,178]. Then, *HvCBF14* has emerged as the major candidate for the frost tolerance in barley in several works [90,96,117,155,178,179,180]. Two SNP linked to *HvCBF14*, associated with frost tolerance, were identified by Fricano and colleagues [178] in an association analysis of a panel of European cultivars, landraces, and *H. spontaneum* accessions. Later on, a correlation between frost tolerance and the same *HvCBF14* gene in spring haplotypes was demonstrated by Guerra et al. [179], who investigated a panel of 403 accessions with exome sequencing-based allele mining.

Structural variation is recognized as a common feature and evolutionary force of genomes, where copy number variations (CNV) and resulting gene dosage effects determine a number of traits/phenotypes in plants [181,182,183,184,185]. One of the first clear associations between CNV and phenotype was reported for the boron-toxicity tolerance in barley [186]. The first indication of the involvement of CNV at the *FR-H2* locus and frost tolerance in *Triticeae* was reported by Knox et al. [121]. Two *HvCBF2* paralogs (*HvCBF2A* and *HvCBF2B*) and multiple copies of the *HvCBF2A-HvCBF4B* genomic segment were identified in the frost-tolerant genotypes ‘Dicktoo’ and ‘Nure’. On the other hand, genomic clones of ‘Morex’ and ‘Tremois’ showed only single paralogs of *HvCBF4* and *HvCBF2.* Results on CNV were confirmed by sequencing the same physical region in the tolerant ‘Nure’ [117] and susceptible ‘Morex’ [116] genotypes, in successive, independent experiments. Francia et al. [122] and Rizza et al. [53] confirmed that frost-resistant varieties of barley were characterized by a high number of copies for the *HvCBF2* and *HvCBF4* genes and maintained two distinct *HvCBF2* paralogs (*HvCBF2A* and *HvCBF2B*). In summary, the influence of structural variation on determining the *FR-2* effect remains a long-standing conundrum and leaves an open question: is the phenotype influenced by the expression of the *HvCBF14* gene alone, or are multiple copies of other *CBFs* involved? Is the number of copies at the *HvCBF2A–HvCBF4B* segment relevant for the modulation of the *HvCBF14* expression level and the resulting phenotype?

In the previous work, Francia et al. [122] evaluated a panel of 41 genotypes using two phenotyping methods (F_v_/F_m_ and field survival) combined with RT-qPCR. The results showed a correlation between the number of copies of the *HvCBF2A–HvCBF4B* segment and frost tolerance. Winter and facultative genotypes showed a higher number of copies and greater frost tolerance compared to spring ones.

The influence of the gene dosage (i.e., the pool of transcripts) of a specific *CBF* on the expression of other elements of the ICE-CBF-COR pathway was tested/evaluated in two elegant experiments. The overexpression of *HvCBF2* in the spring susceptible cultivar ‘Golden Promise’ resulted in higher transcript levels of *COR* genes; *HvCOR14B* and *HvDHN5*, already at warm temperatures, were raised strongly at cold temperatures. Moreover, higher transcription levels of *HvCBF12*, *HvCBF15*, and *HvCBF16* and greater frost tolerance were observed in overexpressed lines [187]. According to authors, *HvCBF2* may activate target genes at warm temperatures, and transcript accumulation for some of them is greatly enhanced by cold temperatures.

The influence of CNV at *HvCBF2A-HvCBF4B* on the expression levels of *HvCBF12*, *HvCBF14*, and *HvCBF16* was investigated using the high frost-tolerant variety ‘Admire’ and different descendent genotypes (namely, Missouri barley—MO B lines) by Dhillon and colleagues [149]. MO B lines harboring a higher number of copies of *HvCBF2A-HvCBF4B* had higher expression levels of all three genes under normal growth conditions.

In addition, Mareri et al. [117] investigated the expression levels and relationship between *HvCBF14* and CNV at *HvCBF2A* and *HvCBF4B* in barley winter, spring, and facultative cultivars with varying degrees of frost tolerance. Authors found higher expression levels for *HvCBF2A* and *HvCBF4B* in winter lines (with a higher copy number) under warm-light conditions. Moreover, putative motifs recognized by other AP2-CBF were identified in the promoters of *HvCBF2C*, *HvCBF12*, *HvCBF12C*, *HvCBF14*, *HvCBF15*, and *HvCBF16*, suggesting an extensive interplay of the *CBF* gene family in response to external stimuli. This observation might indicate that CNV present in frost-tolerant genotypes might play a role in the accumulation of higher levels of transcripts under warm daylight conditions, a kind of “steady-state defense system” ready to react when winter cold arrives. In contrast, *HvCBF14* was inducted by cold in the dark (6 °C), suggesting its role in the activation of the response to a cold stimulus.

These observations, therefore, seem to outline a complex scenario in which the *CBFs* in the proximal-central portion of *FR-2* (phylogenetically classified in subgroup IV) are involved in the response to low temperatures through two putatively complementary and interacting mechanisms: “pre-cold priming” and “cold induction.” The former represents a kind of constitutive *CBF* expression level that fluctuates however, driven by the circadian cycle and light quality and/or intensity. In the case of frost-resistant genotypes, this may lead to the accumulation of higher levels of transcripts due to their higher CNV of *HvCBF2A-HvCBF4B* [117,121,149], and as a result, this may be associated with higher expression levels after the cold stimulus of other *CBFs* and then effector genes [187]. Otherwise, some post-translational mechanisms could be involved that activate the proteins accumulated at “pre-cold priming” only after the cold stimulus [188].

The presence of a basal, light-independent, cold-responsive activation of the HvCBF–COR14B pathway was proposed by Vashegyi et al. 2013 [189]. The expression of *HvCBF14* was shown to be induced by temperature shift and blue light [180], while a significant response to cold, light intensity, and far-red supplementation for *HvCBF14* and consequently the *HvCOR14B* gene was reported [90]. The second mechanism, “cold induction,” is thus based on the major contribution of *HvCBF14* (exhibiting single nucleotide variations) that is up-regulated with the cold stimulus and may regulate effector genes or other *CBF* genes [155,180], leading to cold acclimation via activation of the ICE-CBF-COR pathway. Whether these mechanisms can be effectively exploited for the breeding of superior genotypes remains to be proved and represents an interesting starting point for applied research.

### 2.4. FR-2 in Wheats—CBF Cluster Ploidy

While barley has a diploid genome (2n = 2x = 14, HH) of 5 giga base pairs (Gbp) [190], tetraploid durum wheat (2n = 4x = 28, AABB) has 12 Gbp [191], and hexaploid wheat (2n = 6x = 42, AABBDD) has approximately 17 Gbp [192]. Thereby, *FR-2* organization in wheat is more complex compared to barley due to the contribution of one/multiple homoeologous chromosome regions and redundancy caused by the ploidy level [193,194,195]. Wheat exhibits high variability in frost tolerance traits, given that hexaploid wheat genotypes (AABBDD) exhibit greater frost tolerance than diploid (AA) and tetraploid genotypes (AABB) [196,197].

The first works on *CBF/FR-2* in wheat were carried out in mapping populations of einkorn diploid wheat (*Triticum monococcum* L.), which is the ancestor of the A genome in hexaploid wheat and is considered a practical model for the functional genetics of wheat [75,110,118,119,170,198,199]. First expression studies showed the association of *CBF* genes at the *FR-A^m^2* with the expression of *COR* genes and frost tolerance [75,110].

*TmCBF12*, *TmCBF14*, *TmCBF15*, and *TmCBF16* (central cluster) expression levels were significantly associated with frost tolerance, measured as regrowth capacity after stress. Moreover, a high-density mapping study confirmed that *TmCBF12*, *TmCBF14*, and *TmCBF15* were the candidates for the observed differences [119].

Thanks to the works carried out on *T. monococcum*, the number and position of *CBF* genes in bread wheat were identified in different works. While in barley, a CNV has never been associated with a central cluster at *FR-H2* (see above), in diploid and polyploid wheat, a lower copy number of *CBF14* in the B genome compared to the A and D genomes was reported [200]. Consistent with this finding, Zhu et al. [124] reported that differences in *TaCBF-A14* copy numbers separated susceptible and resistant haplotypes into two distinct panels: winter and spring. Moreover, CNV at *CBF14* in the winter panel was associated with higher expression levels in tolerant haplotypes. *TaCBF-A12* and *TaCBF-A15* were characterized by two insertions/deletions and ten single nucleotide polymorphisms, and CNV was reported. The importance of CNV at *FR-A2* was confirmed by Sieber and colleagues [123] in durum wheat, where approximately 90% of the genotypic variance at *FR-A2* was explained by CNV at *CBF-A14*. Three large deletions, which eliminated 6, 9, and 11 *TaCBF* genes, respectively, were identified in the *FR-B2* locus in tetraploid and hexaploid wheat [193]. These deletions were mainly located in the central part of the cluster, which encompasses *TaCBF-B12*, *TaCBF-B14*, and *TaCBF-B15*, and were observed in cultivated spring wheat, either tetra or hexaploid, associated with lower levels of frost tolerance compared to the wild-type *FR-B2* locus without deletion [193]. Würschum and colleagues [201] evaluated different levels of phenotypic variance in a panel of f 407 diverse European winter wheats, confirming the importance of the CNV of *TaCBF-A14*. In addition, new highly conserved amino acid substitutions in TaCBF-A3, TaCBF-A15, VRN3, and PPD1 proteins were found to be associated with frost tolerance in wheat (*Triticum aestivum* L.) [202].

*TaCBF14* and *TaCBF15* were associated with increased frost tolerance in doubled haploid (DH) mapping populations of ‘Norstar’ × ‘Winter Manitou’ and ‘Norstar’ × ‘Cappelle-Desprez’ (all WH genotypes) [198]. Higher levels of *TaCBF14* induced by temperature shift and blue light were reported in winter wheat ‘Cheyenne’ [180].

Recent studies expanded the investigation of ICE-CBF-COR interconnection with other environmental stimuli with high-throughput functional analysis [78,195,203,204,205,206]. Guo et al. [78] carried out RNAseq and qPCR analysis in wheat tissues under different stress conditions, observing the expression of 53 genes belonging to the ICE-CBF-COR signaling cascade that revealed tissue-specific expression patterns of the *ICE*, *CBF*, and *COR* genes under different stress conditions. Six genes related to the ICE-CBF-COR pathway (*TaCBF11a*, *TaCBF16b*, *TaICE1a*, *TaICE1d*, *TaCOR5a*, and *TaCOR6d.1*) were induced by all treatments (drought, heat, drought, and cold). Three genes, two *CBFs* and one *COR* (*TaCBF1b*, *TaCBF4a*, and *TaCOR3b*), were induced specifically by cold.

Zheng et al. [206] carried out an isoform sequencing experiment at four leaf stages under frost stress (at −6 °C), and expression levels of *TaCBF8a* and *TaCBF14a* decreased, while *TaCBF6a, TaCBF9a*, *TaCBF10a*, *TaCBF13a*, and *TaCBF15a* expression levels increased. Recently, Wang et al. [203] performed a transcriptome analysis during the vernalization (4 °C) time-course with sampling from one to six weeks. Six *CBF* genes of the III subgroup were highly expressed exclusively before vernalization (“steady state” at 22 °C), while 10 *CBFs*, mainly from the IV subgroup, were not expressed before and were highly induced by vernalization, reaching the highest level of expression after three weeks and decreasing after five/six weeks of treatment. Two different homologs of the MYC-like bHLH transcription factor *ICE* were identified in wheat as *TaICE41* and *TaICE87* [146], and their overexpression in Arabidopsis enhanced frost tolerance after hardening. The recent availability of the wheat genome allowed us to locate three *TaICE1* genes on the long arm of homoeologous chromosome group 3; these genes were shown to be induced by drought and cold treatment [78]. In addition, Wang et al. [203] reported that *TaICE41* was expressed at extremely high levels after five weeks of vernalization.

### 2.5. FR-2 in Rye—Evidence of ICE1 Involvement in the Tolerance

Compared to other *Triticeae* crops, rye is uniquely tolerant to biotic and abiotic stresses, showing high yield potential under marginal conditions [207,208,209]. However, it received little attention in terms of breeding efforts and genomic research due to its limited distribution worldwide. Likewise barley and rye have a diploid genome (2n = 2x = 14, RR); however, it has not become a reference crop for genomic analysis in the *Triticeae* tribe due to its elevated level of allogamy and the fact that the first chromosome-scale assembly of its large 7.9 Gbp genome was released only recently, in 2021 [114], showing 92% of repetitive elements [207,210,211,212,213].

Investigation of rye genome evolution and chromosome synteny [213] revealed, as expected, that the chromosome 5R harboring *FR-2* and *FR-1* loci is entirely collinear with wheat homoeologous chromosome group 5. Initially, eleven *ScCBF* genes were isolated in a winter rye genome, and nine of them were mapped on chromosome 5R with a cluster organization (*FR-R2*) [106]. Subsequently, Jung and Seo [207] identified 12 new *CBF* genes and five new *CBF* gene alleles. The genome assembly [114] reported CNV for 4 members of CBF Group IV between tolerant and resistant varieties.

Concerning the structure of the locus, *FR-R2* haplotyped variation has been associated with different frost tolerance levels in different rye genotypes [114,214]. In a recent study aimed at the evaluation of different haplotypes of *FR-R2*, 259 marker-trait-associations (MTAs; *p* < 0.01) were found in 96 genotypes [111]. The ten most significant markers associated with winter frost survival (WFS) corresponded to nine strong candidates, including *ICE1*. Moreover, three MTA identified at a lower significance level matched *CBF* genes at *FR-2R*, namely *CBFIIId-19*, *CBFIVa-2.2*, and *CBFIIIa-6.2*. What is more interesting is that *ICE1* showed 97% sequence identity to orthologous *TaICE41* of hexaploidy wheat, and *CBFIIId-19* and *CBFIVa-2.2* are putatively orthologous to hexaploid wheat genes reported to be induced by vernalization [199,204] (see above). For ICE1 protein, amino acid variation within the DNA-binding bHLH region and/or start of the zipper region resulted in traits such as WFS and low temperature tolerance. Authors hypothesized that the *ICE1* gene identified could be allelic with the *ICE2* gene in rye, whose allelic variation had been reported to be associated with variation for winter hardiness and frost tolerance, and that different *ICE* alleles could be important for frost tolerance in rye [214]. Thus, specific *ICE* alleles (for example, coding for ICE1 proteins with reduced affinity for the MYC binding sites in the promoters of *CBF* and *COR* genes) could be important for winter hardiness [111].

### 2.6. New Frontiers for CBF Genes? CBF Genes in the Drought Stress Adaptative Response

*CBF* genes are members of a large protein family of the C-repeat binding factor/Dehydration responsive element-binding 1 (*CBF/DREB1*), known to be involved in the growth and development processes and responses to different environmental stressors (cold, heat, drought, salt, etc.) [215]. *CBF* genes could thus have in *Triticeae* a role in a cross-talk between the cold and drought response pathways, as already reported for Arabidopsis [98,216].

The *CBF/DREB1* regulon modifies the plant metabolism in conditions of water deficiency, and their activation might also be triggered by drought conditions in the seedling phase [78,217]. In several drought-responsive genes, such as *AtRD29A* (responsive to desiccation), *HvDHN1–HvDHN11* (dehydrin), or *AtCOR6.6*, a DRE/CRT motif is present in the promoter regions. When drought conditions occur, the plant reduces its water uptake by closing the stomata, which also reduces its CO_2_ uptake, which results in a reduction in the photosynthesis and physiological activities. To cope with drought stress, plants activate several morphological and physiological modifications to conserve water and reduce its loss. The molecular response to drought follows a similar pathway to cold acclimation due to the same trigger of water scarcity, which activates both responses. As already summarized (see Figure 1), water deficit activates, like low temperature stress, two different signaling pathways: ABA-dependent and ABA-independent [131]. The interaction between *CBF* genes in hormone-mediated acid abscisic (ABA) pathways has been reported [218,219]. In *A. thaliana*, the ABA-independent pathway is regulated by *AtCBF4*, which increases the production of a class of small, highly expressed, and stress-inducible proteins called late embryogenesis abundant (LEA), protecting the cellular membranes and the cytoskeleton from desiccation [216,220]. Interestingly, it has been shown in *A. thaliana* that *ABA-responsive* genes contain in the promoter regions both the ABA-response cis-element ABRE/ABF and the CRT/DRE motif [221]. Overall, drought stress in barley and wheat can have a significant negative impact on plant growth, yield, and grain quality; however, plants have evolved mechanisms to cope with water scarcity and to survive in dry environments [222]. Nevertheless, which *CBF* genes and which pathways are activated have not been determined yet [88,148,215]. In a study of *A. thaliana* transgenic lines, the overexpression of *AtCBF1* and *AtCBF3* genes resulted in an increase in drought tolerance [223]. A review of the conservative role of *CBF* genes throughout the *Poaceae* family reported rice *OsDREB1A* localized in the cluster *OsDREB1H*, syntenic with the *FR-2* locus on chromosome 5 of *Triticeae* involved in chilling tolerance [96]. However, few examples of studies of the role of *CBF* genes in drought tolerance are available for barley and wheat.

A common phenotypic response observed in transgenic lines overexpressing *CBF* genes in different crops can be identified as an increased tolerance to frost and/or drought and modified growth and development, as originally reported for Arabidopsis [215,223]. Overexpression of two *CBF/DREBs* (*TaDREB3* and *TaCBF5L*) in wheat and barley was reported to lead to an increase in drought and frost tolerance in transgenic barley. Moreover, in transgenic wheat, the *TaCBF5L* gene significantly increased the grain yield under severe drought during flowering [77]. Javadi and colleagues mined available GeneChip microarray data [224] in order to detect key genes involved in drought tolerance in barley and identified hub genes from the AP2 and NAC families that might be among the key TFs that regulate drought-stress response in barley. What is interesting is that *HvCBF6* (distal cluster of *FR-H2*) was included among the hub genes. In rye, the PEG treatment (drought condition simulation) revealed that there is a specific type of response to stress among *ScCBF* genes; most of them were highly responsive to cold stress, whereas *ScCBF2* and *ScCBF7b* were induced by water deprivation and were almost insensitive to low temperature [207]. Guo and colleagues [78] characterized the expression profile of the *ICE-CBF-COR* pathway in different wheat tissues under different stress conditions. Authors showed that *TaCBF11a*, *TaCBF16b*, *TaICE1a*, *TaICE1d*, *TaCOR5a*, and *TaCOR6d.1* were induced by drought, and the induction level was higher in tolerant genotypes [205].

The overlapping of cold/frost and drought conditions is a relatively unexpected new form of combination of abiotic stress, and it usually happens during the late autumn, after sowing, when winter genotypes are in the seedling phase. The drought stress in the seedling phase induces root architecture modification that might act as a constitutive resistance mechanism, useful when the stress re-occurs in other phenological phases [225,226,227]. As observed in numerous studies in the past years, *CBF* overexpression in the model plant Arabidopsis enhances abiotic stress tolerance but, on the other hand, reduces growth. *CBF* genes are known to interact with plant hormones [131], and the current model of CBF-GA (gibberellic acid) interplay proposes that overexpression of *CBFs*, either via cold induction or by transgenic means, stimulates the accumulation of DELLAs. Those growth-repressing proteins act downstream in the GA signaling pathway, leading to stunted growth. As far as the underlying molecular mechanism is concerned, in warm temperatures, DELLAs interact with JAZs to prevent JAZs binding to *ICE1*, leading to its inactivation. In cold temperatures, *ICE1* is modified to gain the function for activation of *CBF* transcription [228]. Understanding the relationship between *CBF* genes, GA and DELLA proteins might help to get an overall picture of the role of *CBFs* in plant physiology. One of the new frontiers that regard *CBF* genes is to evaluate their contribution in the tillering phase, crucial in the growth and development of wheat and barley, as it directly influences the potential yield and overall productivity of cereal crops [229]. Moreover, in this phase, winter cereals reach the maximum of their stress tolerance [28,132,143]. The main actors in tillering formation are gibberellic and abscisic acids; moreover, the roles of *VRN-1* and *VRN-2* and the photoperiod response gene *PPD-1* have been described [230,231,232]. All these components interact with *CBF* genes; however, mechanisms of interaction are still not clear (Figure 4).

## 3. Prospects for *Triticeae* Improvement against Abiotic Stresses

Crop improvement is pursued through different approaches that range from traditional breeding to marker-assisted and genomic selection. Molecular markers facilitate the identification of superior plant materials; however, they still present limitations for complex quantitative traits and in cases of reduced genetic variability. Genomic selection (GS) uses high-throughput genotyping and phenotyping and can be further integrated with the so-called speed-breeding methods and modeling [141], leading to an approach called integrated genomic selection (IGS) [238,239]. Doubled haploid (DH) breeding and the single seed descent (SSD) method can be implemented to reduce the length of breeding cycles and accelerate genetic advances [240,241]. Speed breeding has also been proposed in combination with marker-assisted selection (MAS) for efficient introgression/combination of desired traits in superior genotypes (for a recent review, see [242]). The efficacy of breeding programs can take advantage of multi-dimensional omics; existing germplasm collections can be investigated with the information derived from genome sequences and connected to high-throughput phenotyping data. Incorporating, through climate change models, environmental information sourced from past weather databases or forecasted into the analysis of crop models can yield valuable metadata regarding phenology [238].

Climate change effects that we have been facing in the last decades are increasing the demand for resilient crops that would harbor traits required to tolerate multiple stresses in combination with optimum yield and efficient biomass partitioning [238,243]. The availability of different omics tools (e.g., whole genome sequences, transcriptomes, molecular markers, and linkage maps) could be used to increase and incorporate into new resistant varieties the necessary phenotypic variability [244].

Extensive literature sources regarding the complicated biological pathways involved in the abiotic stress tolerance of *Triticeae* exist. Breeding of wheat and barley is based on their strong autogamous mating systems (outcrossing rates are less than 2% in most situations). For this reason, most new varieties are still obtained via conventional strategies (e.g., inter-varietal crosses, backcrosses, and multi-parent schemes); genotypes harboring the desired traits are artificially crossed, and selection of desirable trait combinations is applied to their progeny. In the case of MAS, functional/diagnostic markers are ideal, in particular when derived from major genes at *VRN-1/FR-1* and *FR-2*. Haplotypes based on multiple markers (mainly SNPs) with strong linkage disequilibrium are more informative and thus were proposed to be applied to target the traits of interest [245], and examples of genome-wide association-based haplotypes were reported in barley [246] and wheat [247]. The contribution of GS (based on prediction of genomic estimated breeding values of individuals modeled from a fully characterized training population) has been also demonstrated in major crops [248]. As the inclusion of functional markers linked to determinant genes can greatly improve prediction accuracy [249,250], molecular markers associated with variation of *CBF* genes could become a useful tool to be integrated in the barley and wheat programs [44,228,251]. Genotypes harboring higher copy numbers at relevant genomic regions within *FR-2* (i.e., *HvCBF2A-HvCBF4B* or *TaCBF14* and *TaCBF15*), in combination with resistant alleles of other *CBF*s (e.g., *HvCBF14* in barley), should be used to create new resistant *Triticeae* cultivars [198,252]. For example, for the development of new, winter malting barley lines, Stockinger [251] suggested advancing a “breeder-friendly” molecular marker approach capable of speeding up the quantification of the CNV in order to integrate it in modern breeding programs [149]. Nowadays, most genotypes used for malting are spring varieties; however due to climate change, in the future, the actual areas of their cultivation, such as US, Canada, UK, Denmark, Germany, Poland, and Hungary, might see the introduction of new winter varieties due to the increasing temperature in the winter [253,254,255]. In wheats, the contribution of homoeologous *FR-2* loci could also be taken into account; a KASP (Kompetitive allele-specific PCR) assay was developed for an SNP at *FR-B2*, which would be included in a set of markers used for breeding and research [256].

Due to the minor worldwide distribution of rye and issues for genetic analyses (given by its cross-pollinator nature with a genetic self-incompatibility mechanism), genomic tools for this species have only been developed recently [257]. Rye genetic resources provide a valuable source of new alleles for the improvement of the species. Although marker-assisted selection was useful for monogenic traits, such as fertility restoration or some disease resistance, only the recent increase in genomic resources has boosted hybrid rye breeding [257,258], giving the possibility to operate on a larger scale than MAS. Rye has strong potential for adaptation to a changing climate, and thus, as far as breeding for frost resistance is concerned, genetic resources should harbor promising alleles for the improvement of this trait in winter elite lines. Whole-genome prediction models assigning a high weight to the *FR–R2* locus allow for increasing the selection intensity through genome-based pre-selection of promising candidates [259].

The above-described approaches proved their efficiency and contributed to developing abiotic stress tolerance in *Triticeae*; however, the process may take years, not in line with the demands of evolving requirements of farmers, growers, industry, and commerce [244]. Therefore, efficient technologies with fast impacts are required to face those issues and challenges [260]. Genome editing enables precise changes in an organism’s DNA by introducing targeted mutations, insertions/deletions, and specific sequence alterations. CRISPR/Cas9 [261] is considered to be the most successful genome editing system for a wide range of organisms [262]. The genes involved in regulatory networks, signal transduction, and metabolite production may be targeted via CRISPR/Cas9 technologies to develop stress-tolerant crops [263]. However, there have been only a few examples of CRISPR-based genome editing approaches in plants for the improvement of abiotic stress tolerance, especially those regarding cold/drought resistance and involving genes from the ICE-CBF-COR pathway. Mutants were generated by CRISPR/Cas9 to characterize the *UGT79B2* and *UGT79B3* genes of the UDP-glucosyltransferase (UGTs) of Arabidopsis, showing that *CBF1* regulates *UGT79B2/B3* and improves stress resistance [264]. *CBF1* was shown, moreover (using CRISPR/Cas9-based *cbf1* mutants), to protect the tomato plant from cold/chilling damage and decrease electrolyte leakage [265]. To augment the plant’s resistance to cold, genome editing was employed to target a few of the transcription factors in rice, e.g., *OsMYB30*, which regulates the amylase gene and negatively affects cold tolerance [266]. The first CRISPR-/ Cas9-based gene editing attempt was conducted in wheat protoplasts targeting *TaDREB2* and *TaERF3*, and the results support the positive regulation of both genes under drought stress and the potentiality of the method [267]. However, it should be taken into consideration that editing single/multiple *CBF*s could not result in the desired phenotype. In fact, an elegant CRISPR/ Cas9 experiment conducted in Arabidopsis clarified the involvement of the *CBF* cluster in the response to multiple abiotic stresses and in plant development [268]. Single, double, and triple mutants in *AtCBF1*, *AtCBF2*, and *AtCBF3* were phenotyped for tolerance to chilling/freezing and to other abiotic stresses and in development. Transcriptome analysis in wild type and mutants after cold induction (4 °C) revealed the complexity of the regulon, with the underlying redundancy and interplay of the genes connected to abiotic tolerance and growth.

## 4. Conclusions

Aiming to improve crops by combining agronomic practices and phenotyping with genetics and biotechnological tools (i.e., molecular markers, QTLs, and haplotype mapping), we reported the latest updates regarding the mechanism involved in abiotic stress response that has the *CBF* genes as the main hub. In this review, the potential role of the ICE-CBF-COR pathway in frost tolerance and the putative involvement in drought tolerance of *Triticeae* were discussed, aiming at increased knowledge on crop growth, stress responses, and tolerance mechanisms. Understanding the structural organization and expression regulation of the *CBF* cluster harbored by the homoeologous chromosome group 5 entails significant potential for genetic improvement of cereals within the *Triticeae* tribe. The retrieval, evaluation, and synthesis of pertinent literature outlined a complex scenario in which the *CBFs* in the proximal-central portion of *FR-2* (phylogenetically classified in subgroup IV) are mainly involved in tolerance to low temperatures. In barley, taken as a diploid model, a mechanism that integrates the “steady state” level of some element(s) under CNV (e.g., *HvCBF2A* and *HvCBF4B*) with a strong induction of other gene(s), among which *HvCBF14* exhibits single nucleotide variations, seems to have a significant role. However, the response/phenotype appears to be governed by two complementary and interacting modes of action, which we called “pre-cold priming” and “cold induced.” The former represents a kind of constitutive CBF expression level that, however, fluctuates driven by the circadian cycle and light quality/intensity. In the case of frost-resistant genotypes, harboring higher copies of the *HvCBF2A-HvCBF4B* segment may lead to the accumulation of higher levels of transcripts at “steady state,” ready to be translated into proteins. Otherwise, some post-translational mechanisms could be involved; CBF proteins accumulated at “pre-cold priming” are activated only after the cold stimulus. The second mode of action is mainly based on the contribution of *HvCBF14*, which is strongly up-regulated by cold, and the final scope of the whole mechanism is a fine-tuning of the CBF regulon, which leads to cold acclimation. Whether this molecular regulation can be effectively exploited for breeding superior genotypes remains to be proven and represents an interesting starting point for applied research. Concerning wheat, the organization of the *FR-2* seems more complex than in barley, due to polyploidy, which appears to confer species-specific levels of resistance. Moreover, specific *CBF*s such as *TaCBF14* and *TaCBF15*, carrying variations in gene copy numbers, have been associated with frost tolerance. Recent studies focused on the ICE-CBF-COR pathway, elucidating an important involvement of the ICE transcription factor that was linked to winter hardiness and frost tolerance in rye. As far as the authors know, no such association has been reported, neither for wheat nor barley, and could therefore explain the greater tolerance of (diploid) rye, even if *FR-R2* hosts a similar number of *CBFs* (per haploid genome). The demonstrated overlapping nature of the adaptive responses to cold and drought, however, appears to be based on the involvement of different CBF/DREB1 factors. While response to cold seems to be driven by subgroup IV, we report works suggesting the potential role of *CBF* members of subgroup III (mapping in the distal region of the *FR-2* cluster) in drought resistance. In conclusion, given the redundant involvement of *CBFs*, an integrated approach based on (i) low-cost markers capable of detecting CNV of key genomic segments, (ii) the introduction of new allelic variants (generated by CRISPR/Cas), (iii) haplotype selection, and (iv) accurate phenotype selection techniques are needed.

## Data Availability

Not applicable.
